# Supplementary material for: COZOID: contact zone identifier for visual analysis of protein-protein interactions
Source: BMC Bioinformatics. 2018 Apr 6;19:125. doi: 10.1186/s12859-018-2113-6 (PMC5889581; doi:10.1186/s12859-018-2113-6)
Supplement: Supplementary file 2 — Software build. Executable binary file of the software tool. (ZIP 118,784 kb) [file 12859_2018_2113_MOESM2_ESM.zip › cozoid/platform/docs/testng-6.5.1-javadoc.zip!/org/testng/TestNGAntTask.TestNGLogSH.html]

TestNGAntTask.TestNGLogSH (TestNG)


---


|  |  |  |
| --- | --- | --- |
| **Overview** \| **Package** \| **Class** \| **Tree** \| **Deprecated** \| **Index** \| **Help** | |  |
| **PREV CLASS**   **NEXT CLASS** | **FRAMES**    **NO FRAMES**     **All Classes** |  |
| SUMMARY: NESTED \| FIELD \| CONSTR \| METHOD | DETAIL: FIELD \| CONSTR \| METHOD |  |


---


## org.testng Class TestNGAntTask.TestNGLogSH

```
java.lang.Object
  org.apache.tools.ant.taskdefs.PumpStreamHandler
      org.testng.TestNGAntTask.TestNGLogSH
```

**All Implemented Interfaces:**: org.apache.tools.ant.taskdefs.ExecuteStreamHandler

**Enclosing class:**: TestNGAntTask

---

``` protected static class TestNGAntTask.TestNGLogSH extends org.apache.tools.ant.taskdefs.PumpStreamHandler ```

---

**Constructor Summary**

`TestNGAntTask.TestNGLogSH(org.apache.tools.ant.Task task,
int outlevel,
int errlevel,
boolean verbose)`


**Method Summary**

**Methods inherited from class org.apache.tools.ant.taskdefs.PumpStreamHandler**

`createProcessErrorPump, createProcessOutputPump, createPump, createPump, getErr, getOut, setProcessErrorStream, setProcessInputStream, setProcessOutputStream, start, stop`

**Methods inherited from class java.lang.Object**

`clone, equals, finalize, getClass, hashCode, notify, notifyAll, toString, wait, wait, wait`

**Constructor Detail**

### TestNGAntTask.TestNGLogSH

```
public TestNGAntTask.TestNGLogSH(org.apache.tools.ant.Task task,
                                 int outlevel,
                                 int errlevel,
                                 boolean verbose)
```


---


|  |  |  |
| --- | --- | --- |
| **Overview** \| **Package** \| **Class** \| **Tree** \| **Deprecated** \| **Index** \| **Help** | |  |
| **PREV CLASS**   **NEXT CLASS** | **FRAMES**    **NO FRAMES**     **All Classes** |  |
| SUMMARY: NESTED \| FIELD \| CONSTR \| METHOD | DETAIL: FIELD \| CONSTR \| METHOD |  |


---
